# A Low Cost Sensors Approach for Accurate Vehicle Localization and Autonomous Driving Application

**DOI:** 10.3390/s17102359

**Published:** 2017-10-16

**Authors:** Rafael Vivacqua, Raquel Vassallo, Felipe Martins

**Affiliations:** 1 Federal Institute of Education, Science and Technology of Espirito Santo, Serra ES 29173-087, Brazil; 2 Department of Electrical Engineering, Federal University of Espirito Santo, Vitória ES 29075-910, Brazil; raquel@ele.ufes.br; 3 Institute of Engineering, Hanze University of Applied Sciences, Assen 9403AB, The Netherlands; fe.nascimento.martins@pl.hanze.nl

**Keywords:** autonomous driving, computer vision, lane marking detector, inertial navigation system, dead reckoning, data fusion, ego-localization, map matching

## Abstract

Autonomous driving in public roads requires precise localization within the range of few centimeters. Even the best current precise localization system based on the Global Navigation Satellite System (GNSS) can not always reach this level of precision, especially in an urban environment, where the signal is disturbed by surrounding buildings and artifacts. Laser range finder and stereo vision have been successfully used for obstacle detection, mapping and localization to solve the autonomous driving problem. Unfortunately, Light Detection and Ranging (LIDARs) are very expensive sensors and stereo vision requires powerful dedicated hardware to process the cameras information. In this context, this article presents a low-cost architecture of sensors and data fusion algorithm capable of autonomous driving in narrow two-way roads. Our approach exploits a combination of a short-range visual lane marking detector and a dead reckoning system to build a long and precise perception of the lane markings in the vehicle’s backwards. This information is used to localize the vehicle in a map, that also contains the reference trajectory for autonomous driving. Experimental results show the successful application of the proposed system on a real autonomous driving situation.

## 1. Introduction

Autonomous driving is the highest level of automation for a vehicle, which means the vehicle can drive itself from a starting point to a destination with no human intervention. The problem can be divided into two separate tasks. The first task is focused on keeping the vehicle moving along a correct path. The second task is the capability to perceive and react to unpredictable dynamic obstacles, like other vehicles, pedestrians, and traffic signalization. This article is focused on the first task and proposes an accurate approach based on low-cost sensors and data fusion, capable of performing autonomous driving.

In order to solve the first task, an autonomous vehicle must be equipped with a set of sensors that allows it to accurately determine its position relative to the road limits. The lateral deviation is the most critical since it must be in the order of few centimeters. The most affordable sensor for direct measurement of position, the Global Navigation Satellite System (GNSS), does not reach this level of accuracy. According to the report [1] of the USA government responsible for the GPS system, the accuracy of the civil GPS system is better than 7.8 m 95% of the time.

There are some special techniques to improve the accuracy of a GNSS system, like Differential GPS (DGPS) [2], Wide Area Augmentation System (WAAS) [3], Real Time Kinematic (RTK) [4]. All those techniques use some kind of correction signal, provided by a base station system, that permits the receiver to calculate its global position with an error of up to few centimeters. For example, the WAAS system was developed by the Federal Aviation Administration (FAA) in order to improve the accuracy, integrity, and availability of the positioning measurement to allow an aircraft to operate autonomously (autopilot) in all phases of flight, including precision approach for landing. In the case of autonomous driving based only on GNSS, even the most sophisticated systems fail. The signal condition in urban environment is strongly degraded due to poor sky view, building obstructions or multi-path reflections. Moreover the correction signal, if available, is not effective in such environment because it cannot compensate a particular degradation condition of an small area. Moreover, a simple GNSS receiver can only be used by an autonomous vehicle to measure its approximated initial position after the autonomous vehicle starts up.

To achieve the stringent level of accuracy, integrity, and availability, required for autonomous driving applications, outstanding projects [5,6,7,8,9,10,11,12,13] have been using widely two kinds of exteroceptive sensors: 3D LASER scanners (LIDAR) [14,15] and cameras. Some of these projects [6,7] also use prior information, for example a map, to compare the current sensor readings, which produces a more accurate, robust and reliable localization. Optionally, these exteroceptive sensors can also be used to detect obstacles, other vehicles, persons, and/or traffic signalization, like traffic light and signs.

LASER scanners, or Laser range finders, are widely employed to detect the road shape or road infrastructures like guard-rails. LASER-based sensors are able to directly measure distances and require less computer processing compared to vision-based techniques. This kind of sensor emits its own light, LASER, and is not dependent of the environment lighting conditions. Another advantage of the LIDAR is its great field of detection. Installed on the roof of the vehicle it covers $360^{\circ }$ around the vehicle so that no other sensor is necessary. For example, the Velodyne HDL-64E LASER range finder has 64 scanning beams that generates a point cloud with hundreds of thousands of points, updated at approximately 10 Hz [16]. LIDARs are being used in several autonomous vehicles prototypes, like the one being tested by Uber [17], and some companies are investing on the development of sensor elements specifically for those type of devices [18]. The drawback of this kind of sensors is that they are expensive and need to be installed on the exterior of a vehicle, often in specific positions, therefore requiring changes in the external shape of the vehicle.

Besides measuring the distance to each detected point, some LIDAR models can give information about the reflectivity of the surface. Some authors [19,20] tested this possibility to recover lane markings painted on the ground, exploiting the different reflectivity between the asphalt and the ink used to paint the lane markings. In [20] a LIDAR sensor was used to measure the reflectivity intensity to detect road markings (lane markings and zebra crossings). This information was used to build a map.

Cameras are very suitable sensors for wide perception of the environment, increasingly being used as a lower cost alternative for the LIDAR. They are generally small [21] and easy to integrate on a vehicle without changing its shape [22]. Besides that, with the exception of some devices built to acquire specific spectral ranges, like long or short wavelength infrared, cameras are quite inexpensive equipment. For example, the camera used in our prototype vehicle costs US$ 25.00 on commercial shops. Another interesting point is that images convey a huge amount of information. Cameras are, in fact, able to acquire texture and color of all framed objects and can also be used to estimate road surface [23,24]. If used in pairs, they can work as a stereo vision system, capable of determining the distance to objects [25].

All those features make cameras very versatile perception devices that can be employed on different tasks. On the other hand, the extraction of this information requires a complex and heavy computational process. Edge extraction, color segmentation, morphological analysis, feature detection and description and object detection, are common tasks performed by computer vision algorithms that require large amount of processing. Therefore, the choice of processing engines have to take into account extra processing tasks for computer vision.

Most of autonomous vehicles based on vision use frontal camera only to detect landmarks. In [26] the frontal cameras are used to detect the preceding vehicle, in order to build the possible lane position. If the vehicle is not present, a lane marking detection algorithm is used. In [27] prior knowledge of the road type is used to trigger two independent algorithms, one to provide the position relative to road geometry, and the other to estimate the shape of off-road path.

Vision-based approaches are also widely used for the detection of road and/or lane markings [28,29,30]. Lane marking detection for lateral localization and curvature estimation is one of the first research fields of computer vision applied to the problem of autonomous driving, since the early 90 s. Many publications and surveys were made [31,32,33,34] describing the diversity of techniques that were developed to solve this problem.

Despite of the limitations, some of the early projects [35] already demonstrated in practice the capability of such systems in following autonomously a road for more than hundreds of kilometers. Actually, if the road has a well painted lane along all of its length, without roundabouts or bifurcations, even a simple system is capable of keeping the vehicle following the road until the vehicle runs out of fuel. More recently, visual lane marking detection regained more attention due to its commercial application in advanced driver assistance systems (ADAS) [36,37,38,39] for lane departure warning and lane keeping systems.

As already mentioned, if a pair of cameras is used, the distance to a detected point can be recovered. So stereo vision can produce the same kind of information as a LIDAR, i.e., point clouds. Despite of the computational power required to process the stereo algorithm, the cost of this kind of sensor is much smaller then one based on LASER scanners. On the other hand, stereo vision is a passive sensor that requires external illumination. Comparing with a LIDAR, stereo vision has smaller field of view and produces less dense information. Even so, some projects of autonomous vehicles use stereo vision to calculate the 3D position of road lane markings. In [40] the position of lane markings in the world is computed using 3D information provided by a stereo camera system using V-Disparity. In [41] 3D features, extracted using stereo vision, are used to create a 3D map in which is possible to self localize using both the real-time computed landmarks and the ones stored in the map.

Other projects of autonomous vehicles use some kind of prior information, like a map, and comparison with the current sensor readings, to increase the robustness and reliability of the autonomous driving algorithm. Autonomous vehicles operating in previously mapped environments [6,7,42,43] have demonstrated the potential of this strategy performing successful autonomous trips in complex real environments. In general a map contains, at least, a set of landmarks used to locate the vehicle and a reference trajectory that is guaranteed to be free of road infrastructure obstacles. If the localization technique achieves sufficient accuracy in any part of the map, the problem of autonomous driving can be reduced to a path following problem.

The localization process relies on the detection and position estimation of these landmarks (see [40,41,44]). Landmarks should not be selected too far from the vehicle since the error in estimating their position increases with the distance. In addition, a landmark should be easy to be detected in order to minimize false positive/negative detections. For example, standard road artifacts like lane markings, stop markings, zebra crossings, or objects like traffic signs and poles are all suitable options. Several approaches use the lane markings as landmarks. Lane markings are easy to detect both by LIDAR and by computer vision. In [45,46] the lane markings are detected by a camera and a computer vision algorithm. In [20], a LIDAR sensor detects the lane markings and the Monte Carlo Localization (MCL) method was used to localize the vehicle in the resulting map with accuracy of 0.31 m.

In [47] digital maps and coherency images were used to estimate the vehicle localization. The maps are constructed from aerial images, where all the information about the lane markings and road surface are manually placed. This map is used to generate artificial images from a given point of view. A particle filter is used to estimate the vehicle position, with the likelihood of each particle being calculated from the coherency value between the current camera view and the artificial image associated to each particle. This system reported a lane-level accuracy of 0.35 m, but has the disadvantage of a manual map construction.

In [44] upright SURF features are used to extract the points which are used for both mapping and localization. This approach is described as topometric, because it is a fusion between topological and metric approaches. The localization is made on a topological map, but the map is geo-localized in order to achieve a metric localization. The method reported an average localization error of 2.70 m.

Now, in [48], image based localization scheme was presented based on Virtual Generalizing Random Access Memory (VG-RAM) [45]. A neural map is built from 3D landmarks, detected by a stereo vision system, and used for localization. The average lateral error reported was 1.12 m, which is not sufficiently small for autonomous driving.

Conversely, in [7], a combination of both front and rear camera systems is used to detect different types of landmarks. The frontal vision system detects road elements painted on the ground (lane markings, stop lines…) to obtain a so called LFL (lane feature based localization). The rear vision system extracts a set of points features to be compared to previously acquired data, to obtain a point feature based localization (PFL). This twofold approach provides a more precise and reliable localization and allows the location system to well adapt to different road conditions of rural, highway, or urban environments. The maps are built offline using images, odometer, and GNSS data collected during a preliminary mapping trip.

### Contribution and Organization of the Text

The main contribution of this paper is the presentation of a light and two-level data fusion architecture using data from a lane marking sensor, a dead reckoning sensor, and a map, to be applied in autonomous driving applications. The lane marking sensor and the map structure are part of the proposed architecture. Mapping is executed using the same set of sensors used in autonomous driving. Another important contribution is the detailed presentation of how low-cost, standard commercial devices, like USB camera, ordinary GNSS receiver, MEMs based gyroscope and a regular notebook computer can be used to achieve accurate vehicle localization and autonomous driving. For instance, we present the design of a Lane Markings Sensor based on a standard USB web-cam, and the study of bias compensation and random noise analysis of a MEMs based gyroscope. Experimental tests show that the accuracy of our proposed system is sufficiently high for autonomous driving operation, even in narrow roads and under challenging lighting conditions.

The paper is organized as follows. Section 2, presents the system overview, Section 3 the visual sensor for lane marking detection, Section 4 describes the proprioceptive sensors architecture developed for dead reckoning, Section 5 presents the data fusion strategy, Section 6 presents the results of accuracy and autonomous driving tests, and, finally Section 7 ends the paper with some final remarks.

## 2. System Overview

**Figure 1:**
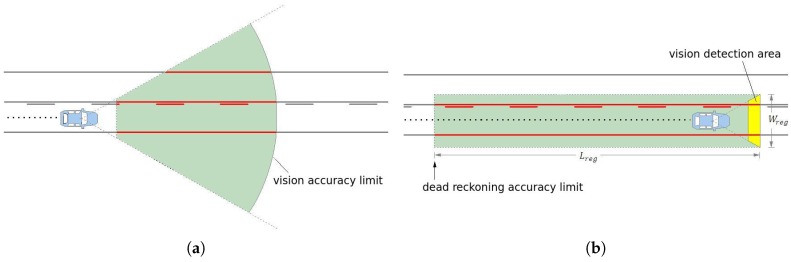
(**a**) Typical medium-range detection limited by the visual accuracy; (**b**) our approach based on short-range detection (yellow) and dead reckoning.

Typical computer vision systems for autonomous vehicles or ADAS (advanced driving assistance), use camera to detect lane markings in the medium range (up to 30 m or 40 m) in front of the vehicle, as shown in Figure 1a. The further away the visual information is from the vehicle, the smaller is its confidence, specially because the variation of the pitch angle, vertical curvature of the ground, or occlusions caused by other vehicles. Thus, the approach presented in this paper avoids this problem by using a visual short-range lane marking detector, and dead reckoning system, that allows to build an accurate and extended perception of the lane markings in the back of the current vehicle’s position. This means that as the vehicle moves, the lane markings detected are displaced backwards so that a back lane mark registry (BLMR) is constructed. The principle, illustrated in Figure 1b, is similar to that used by a document scanner to construct a 2D image from an 1D light sensor array (1D CCD).

Of course that the information in the backwards (BLMR) is not useful to directly drive the vehicle, but when combined with prior information of the environment, like a map, leads to a very reliable and precise autonomous guidance system. In our approach, the BLMR is used to search in the map the current pose of the vehicle (localization). Once the localization is precisely known, and the description of the road is available in the map, the autonomous driving can be performed.

### 2.1. Software Architecture

**Figure 2:**
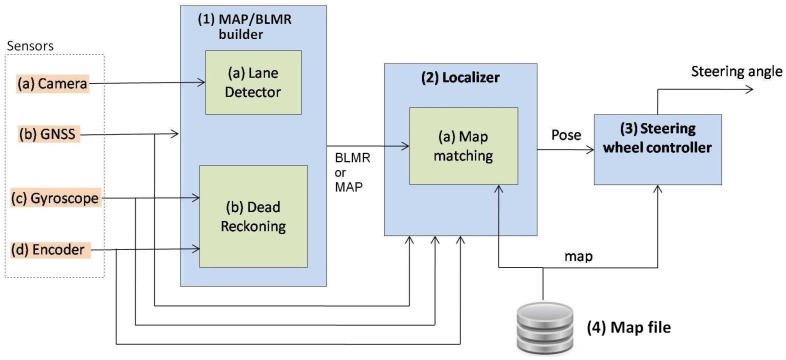
The software architecture for mapping (only Module 1) and autonomous driving (all modules).

Figure 2 shows the sensors and software architecture developed both for map building and autonomous driving. When the system is operating to build a map, only Module 1 is used and its output is a map. When operating in precise localization mode or autonomous driving, all the modules are used. In this case the output of Module 1 is the BLMR. The details will be better explained in Section 5.1.

**Figure 3:**
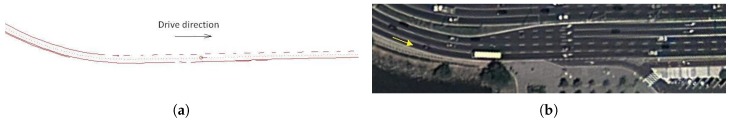
Example of map build by the MAP/back lane mark registry (BLMR) builder module: (**a**) the built lane marking map; (**b**) satellite image of the corresponding road section.

Module 1 fuses information from four sensors to construct the BLMR or a Map, depending on the mode of operation. In reality, the BLMR and a map are the same type of structure, except that BLMR has a limited length and works on its own reference frame. The sub-module (1-a) is the vision algorithm to detect lane markings, and the sub-module (1-b) calculates the dead reckoning. The lane markings detected in the image frame are transformed to the vehicle reference frame by an inverse perspective transformation, corresponding to a bird’s eye view (also called sky-view). The dead reckoning sub-module computes the 2D path of the vehicle (flat world model) based on information from the gyroscope, that calculates the yaw (heading) angle, and the encoder, that measures the linear displacement. Over this 2D path, the detected lane markings are inserted creating a 2D map of the lane marks (or BLMR). One example of this map is shown in Figure 3a, built after the prototype vehicle traveled through the road section of Figure 3b.

**Figure 4:**
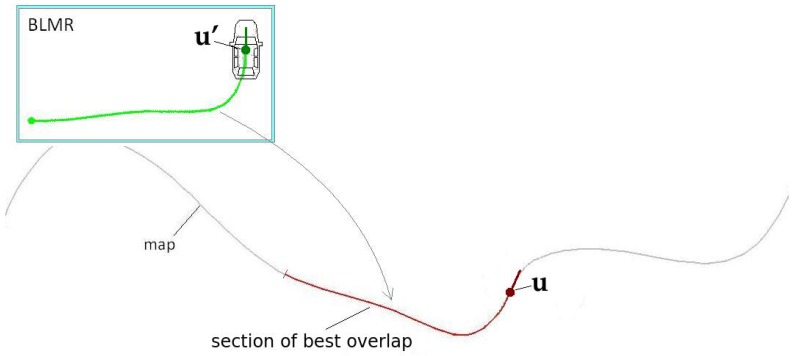
Localization principle: find the map section more similar to the BLMR.

Module 2 is responsible for the localization of the vehicle in the map. The information from GNSS is used only to calculate the approximated initial localization. Once the vehicle knows its approximated localization, and the BLMR data is available, Module 2 applies a technique of map-matching and filtering to calculate the localization with higher accuracy. The principle of precise localization, illustrated in Figure 4, is to search in the map for a section with similar geometric characteristics. As this process is subject to error, additional filtering is applied. The filter also predicts the variation of localization, based on the information provided by the gyroscope and the odometer. Details of the operation of this module are presented in Section 5.2.

Module 3 uses the information of the current localization and road geometry, from the map, to control the steering wheel. Commands are sent to a servo motor that moves the steering wheel to the desired angle. The control rule uses the relative position of the vehicle to a target point, placed in the middle of the road, at a given distance in front of the vehicle. Once this information is known, Module 3 calculates the curvature needed for the vehicle to reach this point. The target point is retrieved from the map and its distance to the car is proportional to the current vehicle’s speed. Details of the control strategy are described in Section 6.2.

Module 4 is a map stored in a file, generated by Module 1, when the vehicle is operating in mapping mode. In mapping mode, the vehicle is driven by a person along the desired path, and at the end, the map is automatically saved in a file. When the vehicle is operating in precise localization or autonomous driving mode, the map file must be loaded to memory to be used by Module 2. Details of the map structure are shown in Section 5.1.

### 2.2. Hardware Architecture

The components that determine the cost of the equipment installed in a vehicle prepared for autonomous driving are the sensors, actuators and the computer system. Among those items, the sensors can be by far the most expensive. In order to achieve a low cost, we avoided the expensive LASER sensor. Instead, we opted for a single camera and a computer vision algorithm for the perception of the environment. Moreover, instead of using a commercial INS/IMU unit, we developed our own, with the minimum functionality, based on low cost inertial sensors and microcontrollers.

**Figure 5:**
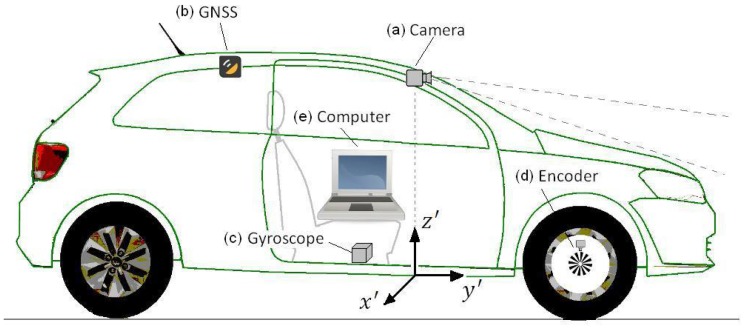
The prototype vehicle and the sensors installed.

Figure 5 presents the prototype vehicle, a VW GOL 1.6, and its hardware setup. This vehicle was used in the first part of the experiments with the objective of determining the localization accuracy. The hardware installed consists of: (a) camera, (b) GNSS receiver, (c) gyroscope, (d) encoder, and (e) computer.

The camera and the vision algorithm forms what we named the lane marking sensor (LMS). The camera we used is a standard USB WebCam (Logitech, model C270) mounted in front of the rear-view mirror for capturing front images. This camera costs about US$ 20.00 on regular computer shops. The vision algorithm processes the image from the camera and calculates the quantity, quality and position of up to four lane markings, within the range of 7.2 m. The algorithm was conceived to be lightweight so that it can run in low cost computers systems. Details of the LMS sensor are presented in the Section 3.

Note that the choice of detecting lane markings close to the vehicle has some advantages. For close lane marks the visual resolution is higher, and the lane markings position computation is less affected by vehicle movements, like pitch and roll, that usually dynamically affect the vision system calibration. The assumption of a flat road, used to estimate the lane markings position, is sufficiently safe when using close lane markings only. Besides that, lane markings close to the vehicle generally are not affected by occlusions. But a short range LMS has a serious disadvantage: it is able to perform autonomous driving only in very limited speeds. Considering a range of 7.2 m, and a reaction time of 1.5 s, this establishes a maximum speed of 17 km/h. This limitation in speed is overcome with a data fusion strategy, explained in Section 5.

The GNSS receiver is a standard low-cost model (UsGlobalSat, model Bu-353-S4) with USB interface and measuring frequency of 1Hz. This kind of receiver, that costs less then US$ 17.00 on commercial shops, was not designed for precision applications. In very favorable conditions, the GPS error of localization is smaller than 7.8 m 95% of the time [1]. This error, that can be much higher in dense urban environments, prevents the use of this kind of receiver in direct autonomous driving. Nevertheless, the value error is sufficient low to be used by the localization algorithm to get the initial estimation of the localization.

The gyroscope sensor is the Invensense model MPU6050. It is rigidly mounted in the bottom of the vehicle to measure its angular speed around the vertical axis. It is constructed from two printed circuit boards. One board contains the gyroscope itself, to measure the angular rate, and the other board contains the microcontroller MSP430G2553, to process its raw signal.

The microcontroller MSP430G2553 is responsible for making the interface between the INS (inertial navigation system) sensor and the computer, to perform some basic calculation and to control the temperature of the sensor. The cost of our mounted sensor is US$5.00, for the INS sensor, plus US$9.00, for the microcontroller board, giving a total of US$14.00. More details of the gyroscope are presented in Section 4.1.

The measurement of the longitudinal displacement (y direction) is done with the original vehicle encoder, used by the speed gauge. So, we had no cost with this sensor. The computer responsible to run the vision, localization and steering command algorithms (see Figure 2) is a laptop with a processor Intel Celeron 1.86 GHz, 1 GB of RAM.

To guarantee that the data received by all sensors is perfectly synchronized we would have to use special sensors, cameras and/or acquisition interfaces that would increase the overall cost of the system. Instead, we choose not to use any special synchronization scheme, and we worked under the assumption that the delay of our sensors is very low with respect to the dynamic characteristics of the controlled system (the vehicle). For example, the vision sensor is the slowest one. We observed a maximum delay of about 30 ms, including frame grabbing, transmission and decompression. If the vehicle is moving at 70 km/h, the longitudinal error corresponds to only 0.58 m, which is a perfectly acceptable value for autonomous driving (note that this is not lateral error). As shown by the results at the end of this article, the overall error of our approach is sufficiently small for autonomous driving.

## 3. Lane Markings Sensor

**Figure 6:**
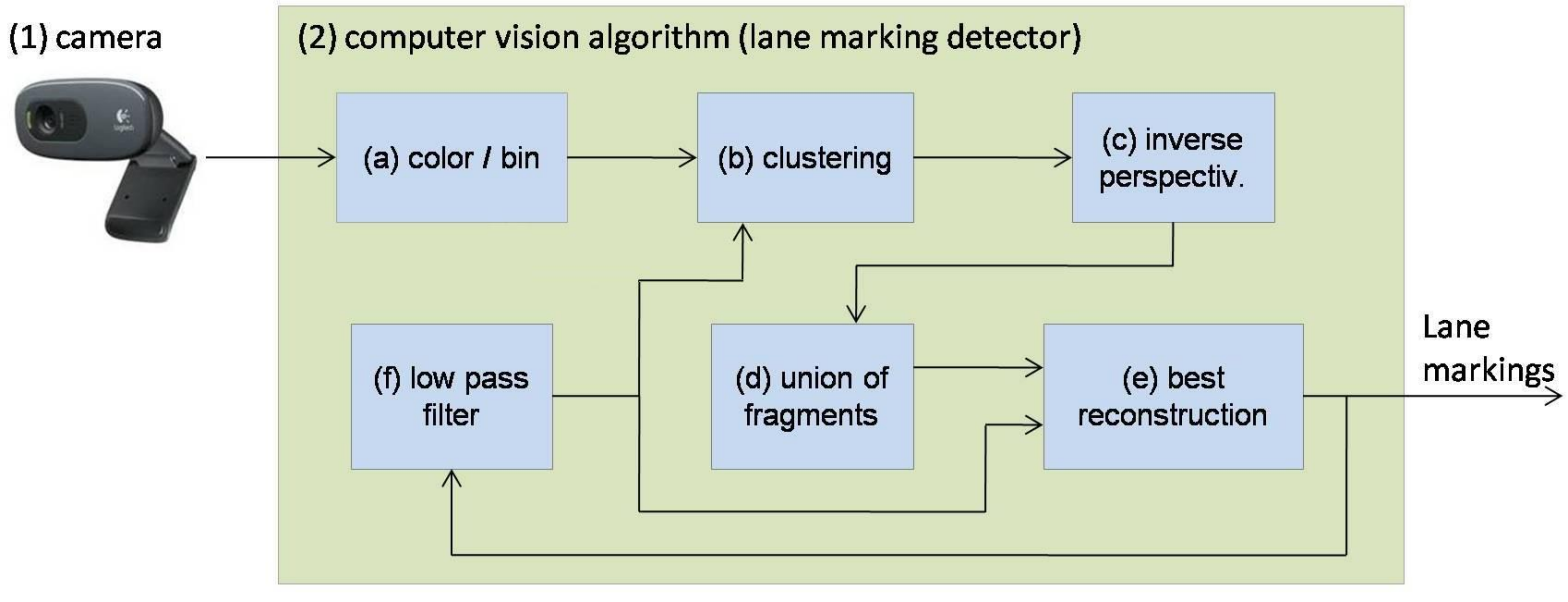
Components of the lane marking sensor: (1) The camera. (2) The computer vision algorithm for lane markings detection.

The lane marking sensor (LMS) is the only exteroceptive sensor necessary for autonomous driving, since the GNSS is used only for initial approximated localization. The sensor can operate both in short range (8 m) or in medium range (30 m). In order to facilitate the explanation of the algorithm, as well as illustrations, it will be considered the operation in medium range. It is formed by a camera and a computer vision algorithm, as shown in Figure 6.

The camera is a standard USB WebCam mounted in front of the rear-view mirror for capturing front images, as presented in Section 2.2. The frames, captured in the resolution of 640 × 480 pixels, are sent to the vision algorithm for lane marking detection. The computer vision algorithm (Figure 6) runs in the same computer of the prototype vehicle together with other processing tasks like dead reckoning, localization, and steering command. Module (a) converts the image from color to gray-scale and then to binary image, classifying the pixels that belongs to a lane mark. Module (b) groups the neighboring pixels. Module (c) calculates, through a perspective transform, the “sky view” of the information, named fragments. Module (d) groups fragments that corresponds to the same lanes makings. Module (e) analyses the result of (d) and selects the best group, according to a quality criteria. Finally, Module (f) filters the instantaneous measurements to feedback a guide line to Module (b). Details of the vision algorithm are presented in the following sections.

### 3.1. Conversion from Color to Binary Image

The conversion from color to binary image is the first step of the vision algorithm and the one that requires the most amount of processing power. Because of the big amount of data, the algorithm must be as efficient as possible. We developed a very lightweight conversion algorithm in two steps: (a) a customized conversion to gray-scale; and (b) a technique of convolution and thresholding. Being lightweight is an important requirement to achieve a low cost sensor for lane marking detection.

#### 3.1.1. Conversion to Gray-Scale

The conversion to gray-scale was customized to enhance the colors mostly used to paint the lane marks, i.e., yellow and white. Instead of using the standard conversion, based on the three color components (Y=0.299R+0.587G+0.114B), we proposed a conversion based only in the color components red and green. The equation used, Y=(R+G)/2, is very fast to be executed, even in processors without float point unity. Moreover, the weight factors highlights the color yellow over the asphalt background, what contributes to the later segmentation of these pixels.

**Figure 7:**
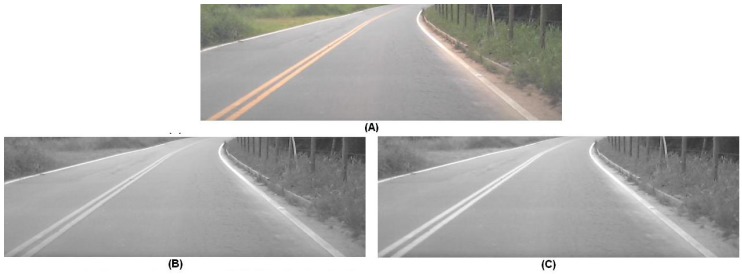
Conversion to gray-scale: (**A**) original image. (**B**) standard conversion. (**C**) proposed conversion.

Figure 7 shows a comparative result of the standard conversion and our approach. The ratio between intensities of the yellow color and the background color (asphalt) increased on average 9% when using our proposed method. This enhanced the capacity of detecting the yellow lanes without changing the response to the white lanes.

#### 3.1.2. Pixel Classification

After the conversion to gray scale the next step is the classification of the pixels that are more likely to belong to a lane marking. This procedure is based on the fact that the lane markings are characterized by being a clear region surrounded laterally by darker regions.

In [49] this condition is detected by a simple and very fast procedure that calculates the difference in brightness between a central pixel and its two neighbors (left and right). A pixel is classified as belonging to a lane if this difference is greater than a threshold value. In [50] this procedure was further improved. Instead of calculating the difference between single pixels, the difference was calculated between the average bright of two groups of pixels, making it less sensitive to noise. The average brightness difference was calculated by convoluting every horizontal line with two step-like mask functions. We used these two ideas in our approach with two improvements: fast calculation of convolution and the use of variable-width masks.

**Figure 8:**
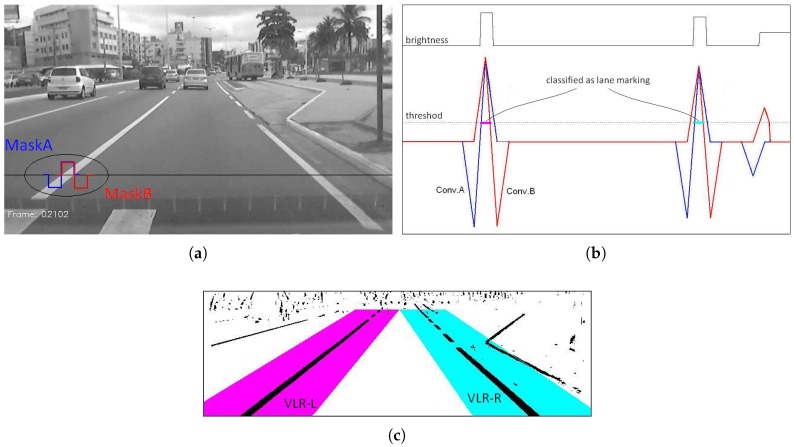
Example of pixels classification. (**a**) Original gray-scale image and masks; (**b**) Processing of a line; (**c**) Final binary image with valid lane regions.

Figure 8a shows the gray-scale image with the two masks used in our approach. The masks have variable width N to compensate for the effect of the perspective projection on the apparent width of the lane marks. For pixels closer to the car the value of *N* is bigger while for pixels towards the horizon line such value decreases. The masks are displaced from each other in half-width (N/2). Figure 8b shows the brightness profile of a single line and, the result of the convolutions with the two masks. In cyan and magenta you see the pixels that are classified as lane marking, that is, those whose values of the two convolutions are above a threshold value. Figure 8c shows the final result as a binary image. The two colored regions, cyan and magenta, correspond to the valid lane region (VLR) for each side, left and right. The VLR cleans up information that is far from the expected position of the lane markings, that is considered noise. The VLR region is molded according to the current state of a low-pass filter.

Convolution is a computationally heavy procedure as it requires a lot of multiplications and sums. For example, for a mask width of 32 pixels, 32 multiplications are needed and 32 sums for each pixel of the processed line. In [50] it was proposed to use integral images [51] to reduce the amount of calculations required for convolution with step-like masks. Nevertheless, 8 sum operations still were necessary for calculating the convolution at each point. If we take into account the sum of 4 operations required to calculate the integral image of each pixel, we have a total of 12 operations.

**Figure 9:**
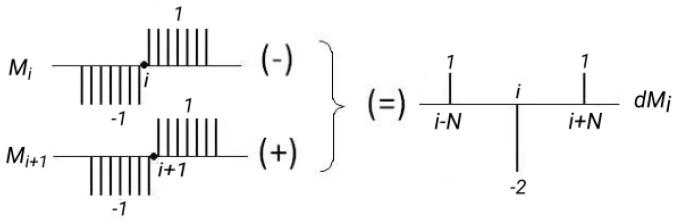
Property of the difference of two discrete step-like functions displaced by one sample.

The algorithm proposed in this paper, calculates the convolution with only 4 sum operations, which makes the classification very fast. The algorithm takes advantage of the property of the difference of step-like functions, illustrated in Figure 9, to calculate the convolution in a recursive manner.

The convolution evaluated for the i-th pixel of a line ($C_{i}$), and its neighbor ($C_{i+1}$), are defined respectively as

(1) $$C_{i}=\sum M_{i}S,$$

(2) $$C_{i+1}=\sum M_{i+1}S,$$

where $M_{i}$ is the step-like mask centered on the i-th position; and S is the brightness vector of a line. Calculating the difference ($C_{i+1}-C_{i}$), and using the property of the difference illustrated Figure 9, we have

(3) $$C_{i+1}-C_{i}=\sum \left(M_{i+1}-M_{i}\right)S=\sum dM_{i}S=S_{i-N}-2S_{i}+S_{i+N}.$$

That results in the recursive expression

(4) $$C_{i+1}=C_{i}+\left(S_{i-N}-2S_{i}+S_{i+N}\right),$$

where $S_{i}$ is the brightness value of the i-th pixel of a horizontal line. The convolution value for the first pixel of a line ($C_{0}$) is calculated normally by Equation (1).

Due to the symmetry properties of the two masks ($Mask_{b,i}=-Mask_{a,i+N}$, see Figure 8a), after the calculus of the convolution with mask A, it is not necessary to calculate the convolution with mask B. The values for mask B can be accessed directly by ($C_{b,i}=-C_{a,i+N}$).

### 3.2. Blobs

**Figure 10:**
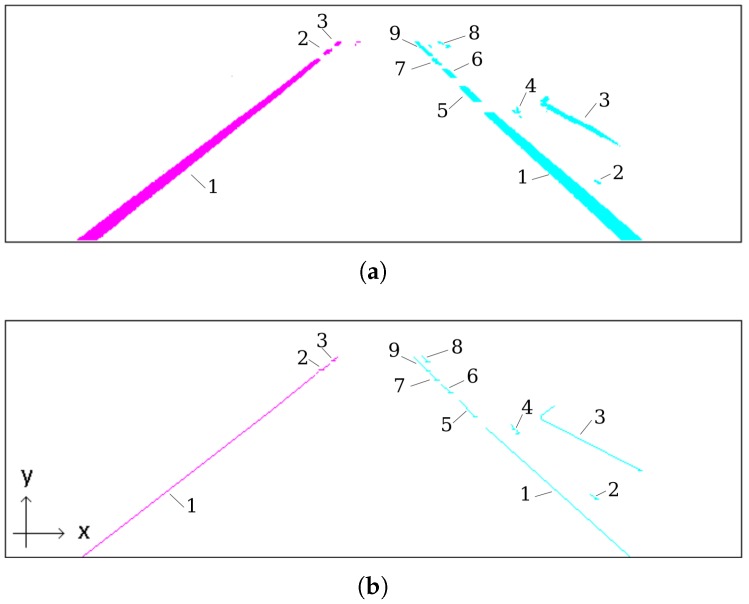
Building the lane marking fragments: (**a**) Blobs. (**b**) Fragments.

After the pixel classification, the resulting binary image, as the one presented in Figure 10a, contains two regions of pixels clusters, one for the left lane marking and other for the right lane marking. Each blob, with a minimum height (Δy) has its mean line calculated to form a fragment (skeleton) representation, stored as an array of pairs (x,y). Figure 10b shows the fragments corresponding to the blobs of Figure 10a.

### 3.3. Inverse Perspective

Each fragment has its points mapped from the image frame to the world frame (x,y)↦(X,Z), using an inverse perspective mapping (IPM), as described by Equations (5) and (6). The transformation projects image points to the road plane, assuming a flat road model,

(5) $$Z=-H_{cam}\frac{\bar{y}\tan\alpha +1}{\bar{y}-\tan\alpha },$$

(6) $$X=H_{cam}\bar{x}\frac{\sqrt{\tan^{2}\alpha +1}}{\bar{y}-\tan\alpha },$$

where $H_{cam}$ is the camera mounting height above the road level; $\bar{y}=\left(y-y_{0}\right)/D_{foc}$ is the value of *y* normalized relative to the focal length of the camera and centered on the optical axis; $\bar{x}=\left(x-x_{0}\right)/D_{foc}$ is the value of *x* normalized relative to the focal length of the camera and centered on the optical axis; and α is the tilt angle of the camera’s focal axis down relative to the horizon.

**Figure 11:**
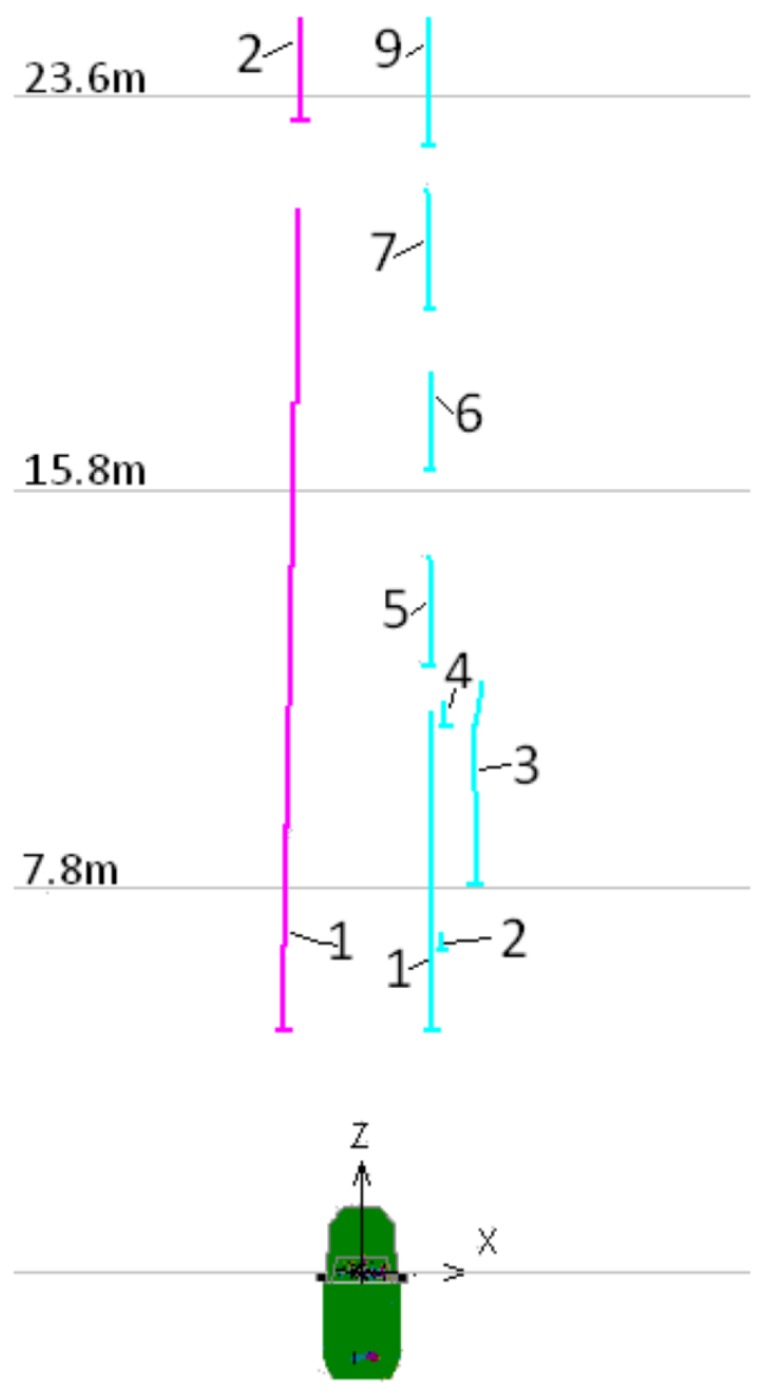
Inverse perspective transformation corresponding to Figure 10 up to 25 m.

The transformation of each fragment generates a new array of points, called mapped fragments. Figure 11 shows the final result after the perspective transformation applied to the Figure 10 up to 25 m.

### 3.4. Union of Fragments

The union of fragments joins separated fragments of the same lane marking, as in the case of intermittent lanes. Moreover, the union of fragments allows to complete gaps that can occur in continuous lanes markings, caused by dust, water puddles, or natural wear of the ink. To speed up the union process, first the fragments are transformed into polynomials. Then a set of tests is performed to determine if two fragments are part of the same lane marking or not.

#### 3.4.1. Conversion to Polynomials

**Figure 12:**
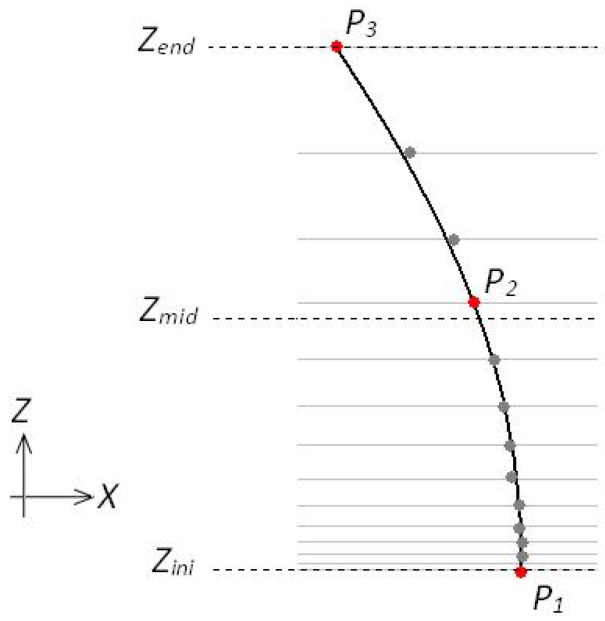
Method for converting the projected fragment into a polynomial.

To represent the geometric information of a fragment, including possible curvature, we chose a second degree polynomial. The polynomial representation is very convenient because it permits fast evaluation of fragments (extrapolation), to determine possible connection with other fragments. The conversion is straightforward and uses three points of the fragment, $P_{1}$, $P_{2}$ and $P_{3}$, to calculate the three coefficients of the polynomial. Figure 12 illustrates the method. Note that due to non-linear transformation of the y↦Z, the increments of *Z* are not linear. The point $P_{2}$ is the point immediately above the center. The coefficients of the polynomial and its interval of validity ($Z_{ini}$ and $Z_{end}$) are stored on a data structure that will be used latter to test for fragments connections, as explained in next section.

#### 3.4.2. Connection Criteria

**Figure 13:**
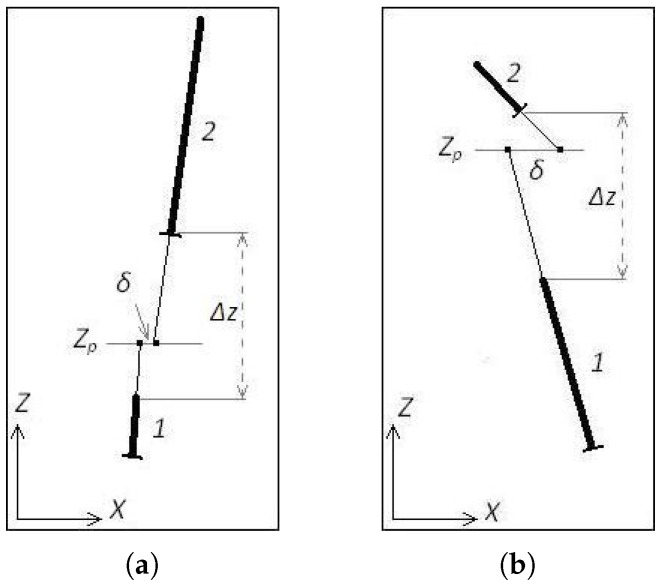
Connection test of fragments: (**a**) Connection established ($\delta \leq \delta_{min}$); (**b**) Connection not established ($\delta >\delta_{min}$).

Two polynomials are connected when there is good evidence that they are part of the same lane marking. To assess this requirement we proposed a set of fast tests, which is based on alignment of the polynomials. Two polynomials are connected when all the following conditions are met:

- There is no overlap between them;
- The smallest fragment is bigger than a minimum length;
- The distance between the fragments (Δz) is less than the sum of their lengths;
- The angular difference between their ends is less than 10${}^{\circ }$;
- The alignment test outlined in Figure 13 is met.

In the alignment test each pair of fragments is extrapolated, using their polynomial representation, until the line Z=Zp according to Figure 13. In Figure 13a, for example, polynomial 1 is shorter than polynomial 2, therefore the line Z=Zp is nearest to the end of polynomial 1 than the top of polynomial 2. Once extrapolated the value of X at the line (Z=Zp), one can calculate the difference ($\delta =X_{1}\left(Zp\right)-X_{2}\left(Zp\right)$). If the absolute value of δ is less than a certain threshold, the connection is established. The threshold value, in meters, was empirically defined as 0.15+0.05Δz. The value of $Z_{p}$ is chosen so that it is proportionately closer to the shorter fragment ($Z_{p}=Z_{1,up}+\frac{L_{1}}{L_{1}+L_{2}}\Delta_{z}$).

**Figure 14:**
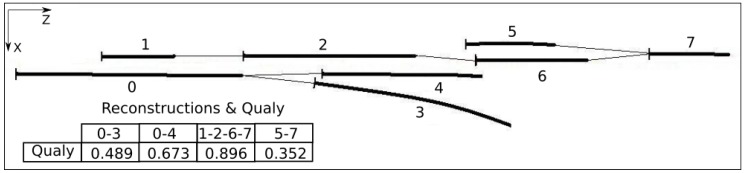
Example of lane marking reconstructions resulting from fragments connection.

After evaluating all possible combinations of fragment pairs, the result is a set of connected fragments. Figure 14 shows an example, in a complex real situation, where seven fragments were mapped. The connection resulted in a total of four possible lane representations. Each reconstruction receives a quality factor (0,0 = min; 1,0 = max) that represents its level of confidence. Note that in most cases, the combination will result in only a single reconstruction. The quality factor is calculated according to Equation (7), and is detailed on the next section.

### 3.5. Selection of Best Reconstruction

The choice of the best reconstruction, among the set of all possible reconstructions (Figure 14), is done by selecting the two reconstructions with higher qualities. If the quality of a reconstruction is lower than 0.25, this connection is abandoned. Different from approaches like [49,50,52], that results in final output information considering only one lane marking for each side (L—Left, R—Right), the algorithm proposed here can use up to four lane markings, being two for each side. For example, in the case of double parallel lanes at the center of the road and a single right track, the extracted visual information of the three tracks will be used, two on the left and one on the right. The use of more lane markings improves the reliability and robustness of the road lateral limit.

**Figure 15:**
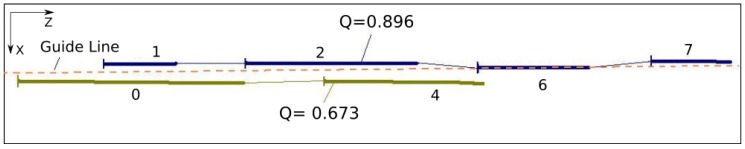
The two best reconstructions corresponding to the example of Figure 14.

Figure 15 illustrates the two best representations corresponding to the example of Figure 14. The dashed line is the guide line, that correspond to the expected position of the lane marking. This information is provided by a low-pass filter, as explained in Section 3.6.

The quality of each reconstruction is calculated by adding the values of quality of each fragment that composes it. In turn, each fragment has its quality calculated in proportion to its length, proximity to the guideline and distance from the vehicle. The heuristic expression proposed in Equation (7) is based on the fact that information between consecutive frames can not vary much and that information coming from more distant points have greater uncertainty. The connections between the fragments are also considered in the computation. Being an artificial complementing of visual information, these fragments receive an extra fee of 20% in the calculation of quality. The quality of a reconstruction is given by

(7) $$Q=\frac{1}{Z_{max}}\sum_{n}^{i=1}\frac{L_{i}}{\left(1+\frac{|\Delta x_{i}|}{\sigma_{x}}\right)\left(1+\frac{|\Delta \alpha_{i}|}{\sigma_{\alpha }}\right)\left(1+\frac{Z_{i,ini}}{0,5Z_{max}}\right)},$$

where *n* is the number of fragments; $L_{i}$ is the length of the ith fragment; $\Delta x_{i}$ is the mean horizontal deviation; $\Delta \alpha_{i}$ is the angular difference; $\sigma_{x}$ is the horizontal penalty parameter; $\sigma_{\alpha }$ is the angular penalty parameter; $Z_{i,ini}$ is the penalty parameter referring to the distance to guide line; and $Z_{max}$ is the range of the vision system. The values of $\sigma_{x}$ and $\sigma_{\alpha }$ were obtained experimentally and 0.50 m and 20${}^{\circ }$ produced good results.

The two best reconstructions, for each side, are transformed in auxiliary polynomials and then fused to create the mixed final representation. Each final representation ($u_{L}$ and $u_{R}$) is defined by the vector coefficients of the second order polynomial $u=(a_{2},a_{1},a_{0})$ and calculated from Equations (8) and (9)

(8) $$u=\frac{Q_{aux1}u_{aux1}+Q_{aux2}u_{aux2}}{Q_{aux1}+Q_{aux2}},$$

(9) $$Q=max(1.0,Q_{aux1}+0.3Q_{aux2}).$$

Equation (8) corresponds to the weighted average for quality. Note that the computation of the final quality from Equation (9) is limited in 1.0.

### 3.6. Low Pass Filter

The low pass filter removes the fast variation of the detected lane markings (output of Module (f) of Figure 6) that contains high frequency noise. The result, named slow variation lane markings (SVLM), is the feedback to Module (b) of Figure 6 which molds the valid lane region (VLR) (Figure 8). The SVLM also is used by Module (e) to compute the penalty of each fragment, as explained in the previous section.

There are two independent filters, one for each side (left and right). Each filter receives as input the coefficients of the final representation polynomial (u) and outputs a new vector with the coefficients polynomial of the SVLM, w. The update is given by

(10) $$w_{i+1}=w_{i}+K_{i}\left(u_{i}-w_{i}\right),$$

(11) $$K_{i}=Q\left[\begin{array}{ccc} 0.16 & 0 & 0\\ 0 & 0.16 & 0\\ 0 & 0 & 0.32 \end{array}\right],$$

where $K_{i}$ is the gain matrix, that varies according to the quality (Q) of the current visual information. When the quality of the detected lane marking is high, the action of the filter is faster. The maximum value of gain, or the maximum response speed, for each polynomial coefficient was defined empirically. The coefficient $a_{0}$ of the polynomial (u) corresponds to the lateral offset of the vehicle relating to the lane markings. When the vehicle is driving not exactly tangent to the road, the value of the lateral offset can vary relatively fast. To accomplish this rapid variation, the update gain for the coefficient $a_{0}$ was chosen to be higher with respect to the others.

## 4. Sensors for Dead Reckoning

Dead reckoning is a technique that allows the estimation of the current position by knowledge of the previous position and integration of speed considering the course [53]. Due to integration, this process is subjected to cumulative error, which limits the range of its application. The cost and accuracy of a dead reckoning system depends strongly on the sensors used, specially the gyroscope. Respecting the low cost philosophy of our approach, we developed a dead reckoning system that uses information from two sensors: the original odometer of the vehicle, used to measure the linear displacement (ΔL) and a low-cost MEMs based gyroscope that measures the angular displacement (Δθ), or yaw rate.

The MEMs (Microelectromechanical Systems) technology [54] combines electrical and mechanical systems at a micro-meter scale. It allows to build moving micro-structures, like accelerometers and gyroscopes, on a silicon substrate. We chose the sensor MPU6050 [55], manufactured by InveSense. It contains a 3-axis gyroscope, a 3-axis accelerometer, and was designed primarily for automotive/commercial performance categories. Precise dead reckoning computation requires the highest level of performance (tactical-grade performance [56]), achieved only by costly sensors. Attempting to extract the maximum performance of our low-cost gyroscope, we took some actions, like temperature control and low frequency noise analysis, as explained in the following subsections.

**Figure 16:**
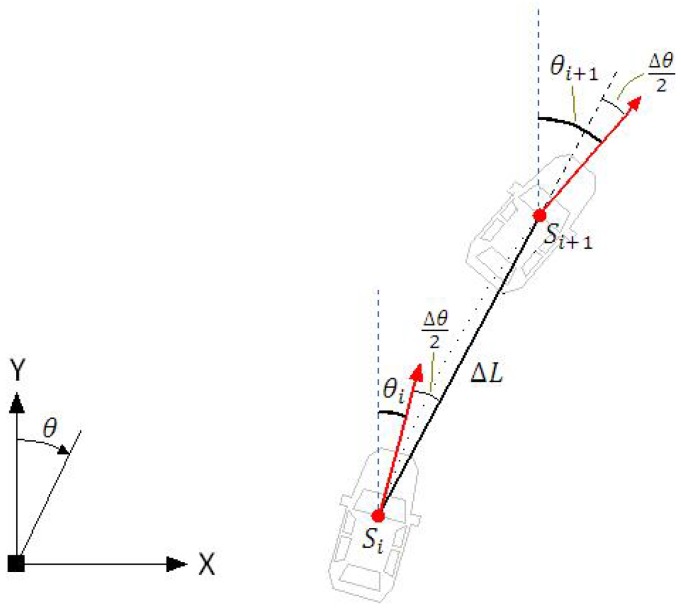
Vehicle’s state reference system.

The dead reckoning computation is performed by the state transition Equations (12)–(14). Given the current vehicle’s pose ($s_{i}={\left[x_{i},y_{i},\theta_{i}\right]}^{T}$), the future pose ($s_{i+1}$) after a linear and angular displacement is computed by

(12) $$x_{i+1}=x_{i}+\Delta L\sin\left(\theta_{i}+\Delta \theta /2\right),$$

(13) $$y_{i+1}=y_{i}+\Delta L\cos\left(\theta_{i}+\Delta \theta /2\right),$$

(14) $$\theta_{i+1}=\theta_{i}+\Delta \theta ,$$

where θ is the yaw angle measured with respect to the y-axis, as illustrated in Figure 16. The equations are executed in the frequency of 25 Hz, which means that at 90 km/h the linear increments will be of 1.0 m. Small linear increments are desirable because they are associated with small angular increments. This allows good description of curves by small straight sections and leads to the simplified Equations (12)–(14).

### 4.1. Measurement of Orientation

The measurement of the angular displacement (Δθ) in Equation (14) is made by integrating the instantaneous angular speed ($\omega_{z}$), provided by the gyroscope, according to Equation (15)

(15) $$\Delta \theta =\int_{t}^{t+\Delta t}\left(\omega_{z}\left(u\right)-\omega_{bias}\right)du.$$

The value of bias ($\omega_{bias}$) corresponds to the value present in the device’s output when its angular velocity is zero. This value should be zero, but it is not for real devices.

**Figure 17:**
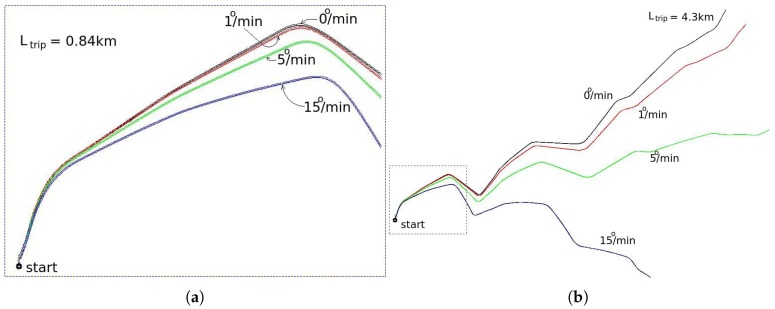
Effect of bias over the track reconstructed by dead reckoning: (**a**) Initial section with 0.84 km; (**b**) Complete section with 4.3 km.

If the bias value is not perfectly compensated, the path reconstruction by dead reckoning will suffer a bend, as illustrated in Figure 17. This figure was generated based on experimental data collected by our test vehicle. The reference path, with no bias, was generated by the GNSS registry, while the others were generated by dead reckoning.

Different values of bias were artificially inserted in the raw gyroscope data collected by our test vehicle, assuming the vehicle is moving at constant speed of 36 km/h. The higher the value of bias, the greater the bend caused in the track. On the other hand, the higher the speed of the vehicle, the smaller the bend because the smaller time of travel.

#### 4.1.1. Gyroscope Bias Compensation

As discussed in the previous section, the precision of 2D path reconstruction by dead reckoning depends strongly on precise bias compensation of the gyroscope. The pure signal generated by a real MEMs gyroscope ($\omega_{z}$) is additionally disturbed by random noise ($\omega_{noise}$) and a constant parcel of that is function of the temperature ($\omega_{bias,T}$).

**Figure 18:**
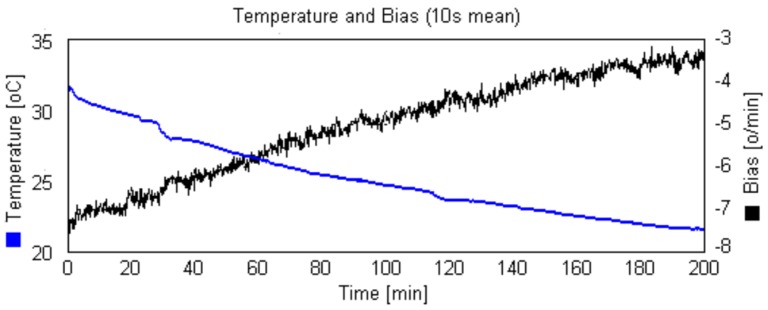
Relation bias × temperature of a tested gyroscope device.

The compensation of the thermal bias component can be done by finding a compensation expression (bias × Temperature). Figure 18 shows the experimental relation obtained for one of our acquired sensor. Note the strong dependence between bias and temperature. For a variation of 10 ${}^{\circ }$C, the bias ranged 8.8${}^{\circ }$/min.

In our approach, we decided to operate the gyroscope in controlled constant temperature. In this case, $\omega_{bias,T}$ becomes a constant and the only remaining source of disturbance is random noise. The constant temperature has the advantage of removing the effect of the temperature over all other gyroscope parameters, like scale factor.

#### 4.1.2. Analysis of Random Noise in Constant Temperature

**Figure 19:**
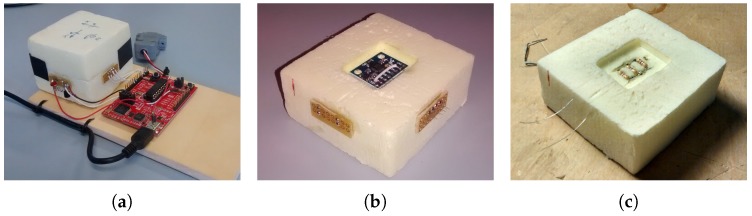
Temperature control for gyroscope: (**a**) control circuit; (**b**) thermal box; (**c**) heating resistors.

To remove the effect of the bias caused by temperature variation, we put the gyroscope to operate with constant temperature. The sensor MPU6050 was fit inside a thermal box, shown in Figure 19, and a heating resistor maintains the temperature in 38 ${}^{\circ }$C. We chose this temperature because it is guaranteed to be higher than the room temperature, but not too much. The temperature control is made by the same microcontroller that interfaces the dead reckoning data to the computer, so no additional hardware is necessary.

**Figure 20:**
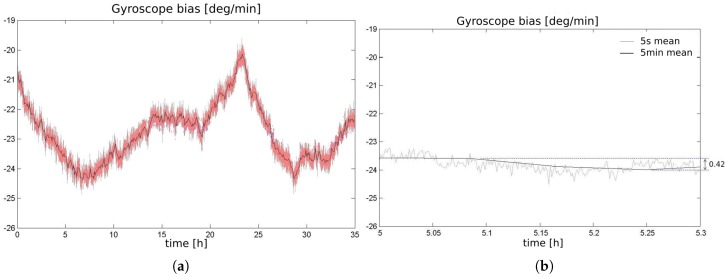
Brown noise characteristic of the gyroscope bias (T = 38 ${}^{\circ }$C): (**a**) long time observation; (**b**) 18 min observation from hour 5 to hour 5.3.

To analyze the random noise of the sensor MPU6050 that we purchased, we collected 35 h of data, in constant temperature, according to orientations of Section 12.11 from the IEEE Standard Specification Format Guide and Test Procedure for Coriolis Vibratory Gyros [57]. The result is illustrated in Figure 20.

MEMs Gyroscopes are subjected to different random noise modes, being predominant the Angle Random Walk (white noise), Bias Instability (pink noise), and Rate Random Walk (brown noise) [57,58]. The Allan variance method is often used to quantify the noise components of a stochastic processes with different properties [58,59].

The yaw angle, computed by the integration of the gyroscope rate signal, is affected by each noise components in a different manner. The white noise is not a problem because its mean value is null and the high frequency characteristics is removed by the integration.

The most critical noise component is the brown noise because of its low frequency characteristics (slow variation). The black line in the graph of Figure 20a, obtained by low pass filtering, illustrates this component. Figure 20b shows that for relative short periods of time, in this case 18 min, the brown noise component presents small variation ($\delta_{b}$), as 0.42 ${}^{\circ }$/min.

**Figure 21:**
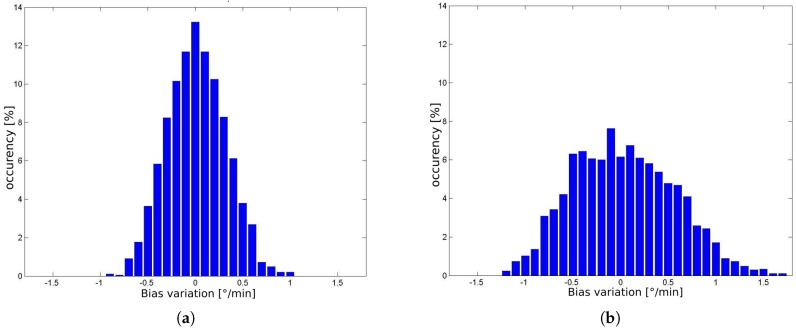
Histogram of the mean bias error caused by low frequency noise: (**a**) for 18 min; (**b**) for 60 min.

Figure 21 shows the histogram of $\delta_{b}$ for intervals of 18 and 60 min. It was computed from 1000 randomly taken sections of signal of Figure 20a (black line). When the localization system of the vehicle starts-up, first it measures the current value of the bias, by averaging the gyroscope signal for 15 s. So, the histogram of Figure 21 permits to calculate the probability of this initial value to drift by a certain amount, for the next period of time (18 and 60 min).

As introduced in Section 2.1, the principle of localization is the comparison of the shape and content of lane markings of small road segments. Figure 17a illustrates that bias drifts in the range of 1 deg/min, causes very small distortion in the shape, and consequently, we assume that it doesn’t compromise the localization algorithm to work properly. The histogram of Figure 21b together with the consideration of the prior phrase, are a strong indicative that is possible to operate the autonomous vehicle properly up to 60 min or even more. After this time limit the vehicle must stop to measure again the bias value. Another possibility, is to update dynamically the bias, using information from the GNSS receiver, since it is not subjected to accumulative error. This is one of the issues addressed as future work.

A deeper comprehension on how the distortion caused by the bias error affects the accuracy of the localization is not a simple task. It depends on the particularities of each road section, like shape and distribution of the lane markings information over the BLMR. This is also planned as future work.

### 4.2. Measurement of Linear Displacement

The measurement of linear displacement is made directly by the pulse count ($N_{p}$), given by the equation $\Delta L=k_{e}N_{p}$. The pulse signal is taken from the original encoder of the vehicle, used by the speed gauge, and connected to the same microcontroller board used by the gyroscope. The constant $k_{e}=0.2674$ is determined by the tire diameter and the number of pulses generated at each complete revolution of the wheel.

## 5. Data Fusion

**Figure 22:**
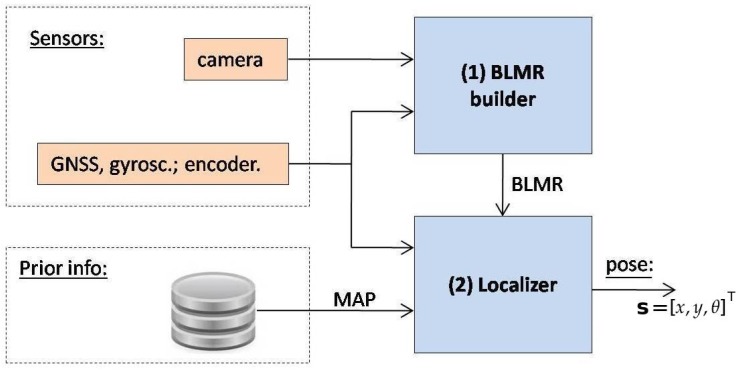
Data fusion strategy for vehicle’s pose estimation.

Trying to achieve the maximum accuracy of localization within the road, the information of the four sensors is fused with the prior information of road geometry and lane markings. According to Figure 22, the fusion is performed in a cascade scheme: (i) first, all sensors are used to construct the back lane marking registry (BLMR) and, (ii) second, BLMR, MAP, GNSS, gyroscope and encoder are combined to estimate the vehicle’s pose in the map reference frame.

**Figure 23:**
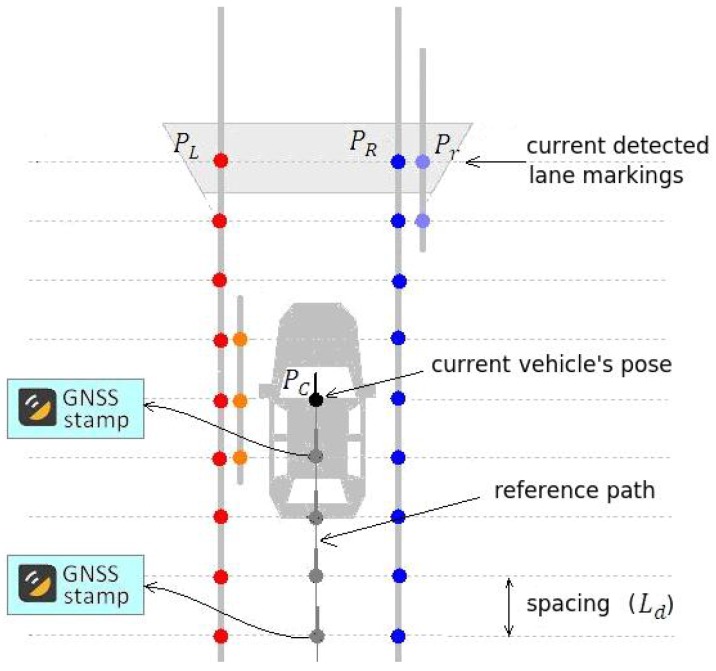
Data structure: Sampling the i-th position and lane markings information in the longitudinal discrete space.

The prior information consists of a map that contains a reference path and the detected lane markings. A map is built by the prototype vehicle being manually driven across the desired path. The collected data, whose structure is presented in Figure 23, is stored in a file to be used latter during localization and autonomous driving.

### 5.1. First Level Fusion: BLMR

Module 1 of Figure 22 fuses information from the gyroscope, odometer, vision and GNSS, to build a 2D map of the lane markings (back lane marking registry—BLMR) on a proper reference frame. The difference between the BLMR and the map, stored in a file, is that it has limited length and the origin is always in the current position of the vehicle. Thus, for simplicity, we will refer to the output result of Module 1, simply as BLMR. The BLMR is the result of the first level fusion of the four sensors.

The gyroscope and the odometer are combined for dead reckoning (Section 4) to build the 2D path driven by the vehicle. This path, named reference path, corresponds to the points covered by the center of the vehicle. The reference path of a map is used as a guide path for autonomous driving, as will be discussed in Section 6.2.

Over the reference path, the visual information (lane markings) and the GNSS information (global position) are inserted. The lane markings detector finds the position of the lane markings in the image coordinate frame. This information is transformed to the vehicle reference frame (x’/y’) by an inverse perspective transformation, corresponding to a bird’s eye view, while considering the camera in the origin. Now, changing from the vehicle coordinate frame to the map coordinate frame is made by a rotation and translation operation. Any point of a lane marking ($P_{lane}^{\prime }$) in the vehicle coordinate frame has its corresponding position ($P_{lane}$) in the map coordinate frame computed by

(16) $$P_{lane}=P_{c}+R_{\theta }P_{lane}^{\prime },$$

where $P_{c}={\left[x,y\right]}^{T}$ is the vehicle position at the time of capturing the image, $R_{\theta }$ is a rotation matrix around the vertical axis by an angle θ, and θ is the vehicle orientation (heading) at the time of capturing the image.

#### Details of Data Structure

Both the BLMR and the map are built from a discrete set of samples uniformly spaced along the direction of the vehicle movement, as shown in Figure 23. This discretization simplifies and speeds up the point association during the map matching. Ideally, the increment value ($L_{d}$) should be as small as possible, but that would generate a very large amount of points that makes the localization process heavier. A value of $L_{d}=$ 1.33 m was considered a good compromise between accuracy and execution speed.

Each map sample contains information of the reference path (P) and the position of up to four lane markings ($P_{L},P_{l},P_{R},P_{r}$). The quality factor of the detected lane marking is copied to the lane point. Missing tracks are registered with a zero quality value, and well detected tracks have quality close to 1.0. The quality attribute is used in the map matching process to compute the confidence of the pose measurement.

Every time the GNSS receiver reports its current position, the information of latitude and longitude is associated with the last sample inserted on the map as a GNSS stamp. When running the online localization algorithm, this connection allows the vehicle to determine its starting location based on the current position given by a GNSS reading.

### 5.2. Second Level of Fusion: Localization

The second level of fusion, performed by Module 2 of Figure 22, is made by a scheme of filtering that uses information from the GNSS and from the BLMR to update the estimation of vehicle’s pose, $s={\left[x,y,\theta \right]}^{T}$ in the map. The filter considers the uncertainty of each method during the update, and uses the information from the proprioceptive sensors (gyroscope and odometer) to predict the incremental displacement of the vehicle (next localization).

The measurement of localization using information from the GNSS receiver is made only in the start up process, to calculate the approximated initial localization of the vehicle, while the BLMR is still empty. After the BLMR stores sufficient information of the lane markings in the backward vicinity of the vehicle, the filter ignores the GNSS and starts to use exclusively the BLMR as information to update the localization state. Then, the measurement of localization is made by a technique of map-matching between the BLMR and the MAP. The obtained accuracy is sufficiently high to permit autonomous driving mode as shown in Section 6.3.

#### 5.2.1. Pose measurement by GNSS

After the system starts up, the first estimation of localization is made from GNSS information. The localizer module uses the current global position reading to search, in the map, the GNSS stamp that has the shortest euclidean distance to it. Once the closest stamp is found, the system checks whether its distance is below a minimum value, to guarantee that the car, is in fact, searching in the right part of the map. If so, the localizer uses the pose of the vehicle associated with this stamp to set its initial pose ($s_{0}=p_{c,k}$).

This initialization strategy assumes that the vehicle is in fact within the mapped road and correctly oriented in the route. The accuracy of this localization is too low to allow autonomous driving, but it is sufficient to define the initial search region for the BLMR pose measurement algorithm, as described in next section.

#### 5.2.2. Pose Measurement by BMLR Overlap

After the vehicle starts to move, the BLMR starts to accumulate visual information about the lane markings. When the quantity of information reaches 75% of the nominal operating value (240 m), the measurements for localization are now performed by map-matching between the BLMR and the MAP. The BLMR corresponds to a short map of the backward vicinity of the vehicle, composed by an array of samples that contains the vehicle poses and the information of the lane markings detected.

**Figure 24:**
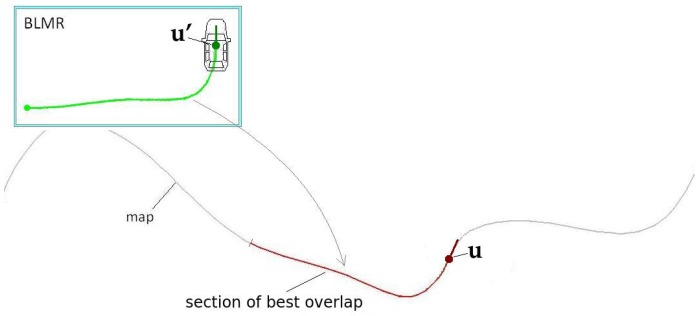
Localization principle: find the map section more similar to the BLMR.

The principle of pose measurement by BLMR overlap is to find the transformation (translation and rotation) that, applied to the BLMR, results in the best overlap of the lane markings from the BLMR and the map (Figure 24). Once this transformation is found, named best match transform (BMT), it is applied to the current pose of the vehicle ($u^{\prime }$), in the BLMR reference frame, to obtain the pose measurement (u), in the map reference frame. As this process is subject to noise, the pose measurement is filtered and the result is a pose estimation (s).

The localizer (Module 2 of Figure 22) searchs for the BMT starting from the point in the map (point $M_{0}$) that is closer to the current vehicle position (s). Six other map candidate points are also evaluated, three points ahead and three points behind $M_{0}$. Figure 27 illustrates such points.The candidate point that produces the best overlap (minimum matching error) is selected. If the map-matching error is small, this means that the recent sensor readings, registered in BLMR, are consistent to a given position on the map. Therefore it is likely that this point is near to the true position of the vehicle.

The matching error adopted in this work ($\varepsilon_{m}$) represents the average value of the lateral difference (absolute value) between the samples of the transformed BLMR lane markings and the map. Thus, the lower the value, the more similar are the BLMR and map information. The matching error is calculated according to Equation (17). We propose the use of a weighting function, $G_{k}$, to take into account the uncertainties inserted by the dead reckoning: the farther back is the sample, the higher is its uncertainty. The proposal function to penalize these points is a Gaussian $G_{k}=exp\left(-{\left(\frac{k}{n}\right)}^{2}\right)$, where *n* is the number of BLMR samples (*n* = 180). The matching error is given by

(17) $$\varepsilon_{m}=\frac{\sum_{k=1}^{n}G_{k}\sum_{i=[R,r,L,l]}Q_{map,i,k}Q_{blm,i,k}\left|\delta_{i,k}\right|}{\sum_{k=1}^{n}G_{k}\sum_{i=[R,r,L,l]}Q_{map,i,k}Q_{blm,i,k}},$$

where $Q_{map,i}$ is the quality of the i-th map sample; $Q_{blm,i}$ is the quality of the i-th BLMR sample; and $|\delta_{i,k}|$ is the absolute value of the lateral difference between the BLMR’s and the map’s lane marking points.

**Figure 25:**
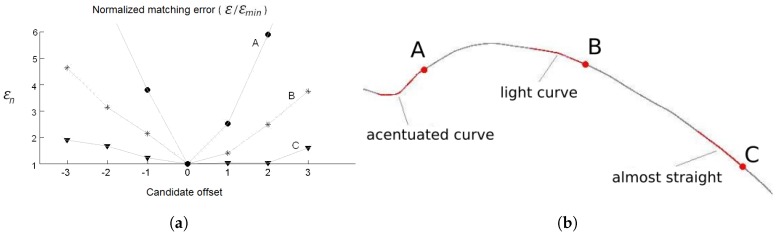
Relation between the BLMR shape and the matching error. (**a**) matching error graph; (**b**) different BLMR shapes.

The choice of the best candidate point (BMT) is performed by analyzing the matching error. This can be seen as a longitudinal localization process. The chosen candidate point is the one with the lowest value of matching error, as shown in Figure 25. This figure, generated from real data collected by our test vehicle, illustrates the search algorithm operating in three different conditions: (A) a sharp curve; (B) a light curve; and (C) a section that is almost straight. Note that the election of the best candidate point in case (A) is more reliable then in case (C). In other words, we can state that the longitudinal uncertainty of the pose measurement is affected by the shape of the BLMR.

The more straight is the BLMR, the higher is the uncertainty of the longitudinal pose measurement. When the BLMR section is nearly straight, the value of the matching error presents a small variation for all candidate points (Figure 25b, case C). This means that it is not possible to achieve a precise longitudinal localization in this case. On the other hand, if the BLMR section contains sharp curves (Figure 25b, case A) it will produce a steeper response in the graph of the normalized error (Figure 25a, case A), which increases the confidence on choosing the best candidate point (longitudinal localization).

**Figure 26:**
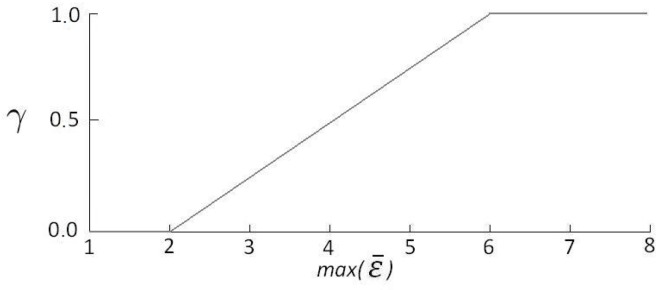
Confidence parameter for longitudinal measurement.

To consider the longitudinal uncertainty of the pose measurement in the filtering process, we propose, based on the graph of Figure 25a, the mathematical relation between the maximum value of the normalized matching error ($max(\bar{\varepsilon })$) and the confidence parameter (γ), illustrated in Figure 26. We established two threshold values for $max(\bar{\varepsilon })$. When it is smaller than 2, we considered that the BLMR is a perfect straight line, i.e., the confidence parameter is null. When it is greater than 6, we considered that the BLMR contains sufficient curvature so that the confidence parameter is maximum. The confidence parameter (γ) is used by the filter to adjust the longitudinal update gain, as explained in next section.

#### 5.2.3. Filter

When the system starts up there is no filtering action, and the localization state is assumed to be equal to the last measurement provided by the GNSS (Section 5.2.1). After the BLMR is ready to be used, the filter enters in operation to give more robustness to the pose measurements by BLMR map-matching (Section 5.2.2).

**Figure 27:**
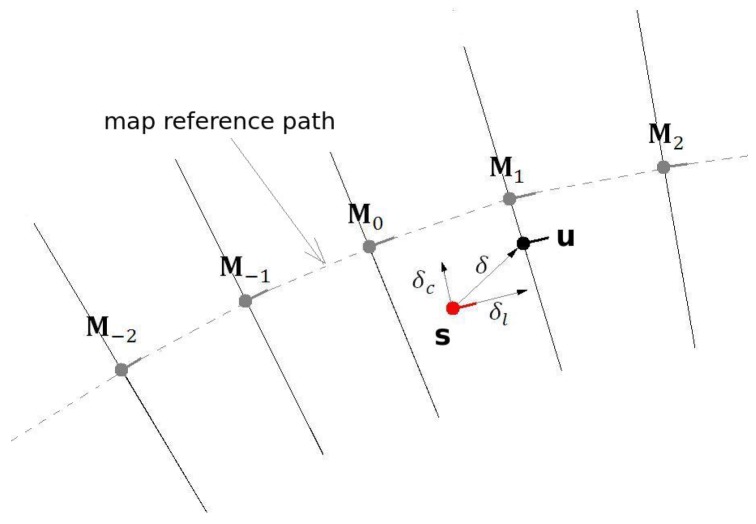
Domain of pose measurements: the black lines are the places that a measurement **u** can occur and **s** is the current vehicle’s pose.

As any measurement process, the pose measurement by BLMR map-matching is susceptible to noise. Particularly, in this case, the way the noise affects each component (longitudinal, lateral, heading) is different. In Figure 27, the gray dashed line represents the map reference path. Each measurement, $u=(x_{u},y_{u},\theta_{u})$, can be decomposed in a discrete longitudinal component, due to the process of selection of BMT, a continuous lateral component and a continuous heading component. The gray lines in Figure 27 illustrate the places where the measurements may occur. Note that the current vehicle pose, **s**, is not subjected to this restriction.

To reduce noise effect in the measurements produced by BLMR map-matching, given the particularities of each component, we used a low pass first order filter with independent action on each component. For the lateral deviation ($\delta_{c}$) is used a constant gain ($k_{c}$), for the longitudinal deviation ($\delta_{l}$) a variable update gain ($k_{l}$), and for the heading deviation ($\delta_{\theta }$) another constant gain ($k_{h}$). The update of the vehicle state is done by the Equations (18) and (19), every time that a new sample is introduced into the BLMR.

(18) $$s_{i}=s_{i-1}+R_{\theta }KR_{\theta }^{T}\left(u_{i}-s_{i-1}\right)$$

(19) $$R_{\theta }=\left[\begin{array}{ccc} \cos\theta & -\sin\theta & 0\\ \sin\theta & \cos\theta & 0\\ 0 & 0 & 1 \end{array}\right],K=\left[\begin{array}{ccc} k_{c} & 0 & 0\\ 0 & k_{l} & 0\\ 0 & 0 & k_{h} \end{array}\right]$$

where *K* is the gain matrix for each component, $R_{\theta }$ is a rotation matrix for decomposition of the lateral and longitudinal components of the deviation.

As discussed in Section 5.2.2, the longitudinal localization is a process whose uncertainty, or random error, depends on the shape of the BLMR. To consider this variable uncertainty in the filtering process, we proposed the use of the confidence parameter γ (see Figure 26) that acts directly in the longitudinal gain of the filter according to

(20) $$k_{l}=\gamma k_{lmax},$$

where $k_{lmax}$ is the maximum value of longitudinal filtering gain.

When the BLMR is almost straight, the confidence parameter γ is null, so no update in the longitudinal localization will be done. Instead, when the BLMR contains good curves, the confidence parameter γ will be nearly one, so the update will be performed with the maximum value of gain ($k_{lmax}$).

The longitudinal measurement is the most noisy component due to the discretized longitudinal space (Figure 27). To properly attenuate this strong noise it is necessary to use a low value for the maximum gain ($k_{lmax}$), or a slow filter action. A slow update action over the longitudinal localization is not a problem because the longitudinal localization is much less important than the lateral localization to keep the car in the center of the road, when performing autonomous driving. The discretization noise could be reduced by using a smaller longitudinal resolution, but this would increase the number of points used in the process of matching and thus the processing time.

The lateral component is the most critical information when keeping autonomously the vehicle in lane, so a fast response is required. Moreover, the lateral measurement, performed by the short-range lane marking detector, produces small values of error. Because of this, the gain for this component must be much larger then for the longitudinal component.

Regarding the heading gain, heading measurements generated by the BLMR localization module are quite stable and, therefore, a fast filtering action can be used, just like for the lateral component.

The initial values for the gains were set to $k_{c}=0.1$, $k_{l}=0.01$, and $k_{h}=0.1$. These values were then fine tuned during experimental tests and the best results were achieved with $k_{c}=0.25$, $k_{l}=0.008$, and $k_{h}=0.25$.

The filter also uses information from the dead reckoning sensors (Section 4) to update the filter state, according to Equations (12)–(14).

## 6. Tests and Results

The proposed localization system was built with the low cost sensors explained in Section 2, Section 3 and Section 4, and by performing data fusion detailed in Section 5. Its evaluation was performed in two parts. The first part of the tests was made in Brazil, with the objective of analyzing the error in the information required to perform autonomous driving (Section 6.2).The second part of the tests was made in Italy, with the objective of verifying, in real traffic conditions, the performance of autonomous driving based on our localization method. At the end of this section we also present the computational performance of the algorithm.

### 6.1. Performance of the Dead Reckoning Sensors

**Figure 28:**
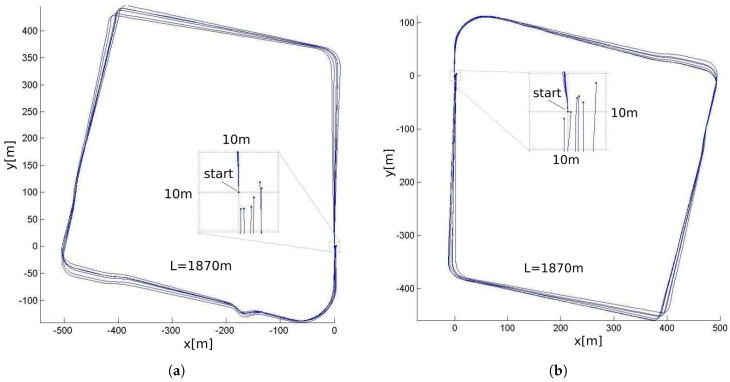
Evaluationof the dead reckoning sensor on a closed path: (**a**) clockwise direction; (**b**) counterclockwise direction.

The performance of the dead reckoning sensors was evaluated through a test on a closed path of 1870 m. The test vehicle, equipped with the encoder and gyroscope, was driven manually starting and stopping on the same real position, painted on the asphalt. In this kind of test the initial orientation is not critical and the error can be accessed directly at the final position. We performed a total of 12 trips, 6 in each direction (clockwise and counterclockwise). Before each lap, the initial value of the gyroscope bias was measured, by averaging the raw signal during 10 s. The result is presented in Figure 28. The mean value of the obtained error was 2.4 m (0.13%) and the maximum, 5.3 m (0.28%).

### 6.2. Localization Accuracy

The localization system presented in this paper was designed mainly for autonomous driving purpose, which is one of the applications that require the highest level of localization accuracy. Thus, the methodology used to calculate the localization accuracy considered the information that directly affects this objective.

**Figure 29:**
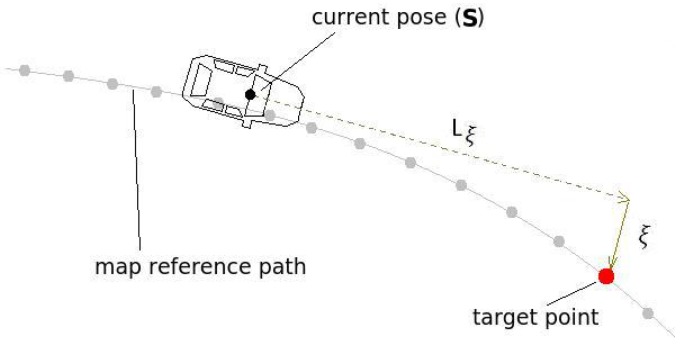
Autonomous drive based on following a forward target point.

Module 3 in Figure 2 is responsible for computing the steering angle. This module uses information from the vehicle’s current pose and the map to calculate the lateral deviation from the lane’s center, with respect to the vehicle longitudinal axis (ξ), and compute the necessary steering angle to keep the vehicle on the reference path. This principle is shown in Figure 29 and was used in [35,60].

The radius necessary for the vehicle to reach the target point is calculated by Equation (21)

(21) $$R=\frac{\xi^{2}+L_{\xi }^{2}}{2\xi }.$$

For a given distance $L_{\xi }$, the lateral deviation ξ can be accessed from the vehicle current state on the reference path obtained from the map (Figure 29). Note that the only variable susceptible to error, that can transfer error to the calculation of the radius, is the variable ξ. For this reason, we proposed a method to evaluate the localization accuracy based on the analysis of the lateral error of the target point (LETG), that is, the error of ξ. Therefore, to perform such evaluation, a ground truth reference is needed. In our case the error is calculated with respect to a considered-ground-truth (CGT) reference. More details on how the CGT is calculated are given in [61].

The distance $L_{\xi }$, used in the analysis, is fixed and was chosen considering the speed of 60 km/h for the mapped section, and the time-to-reach of 1.5 s. The target point is taken directly from the map reference path (Figure 29), choosing the one whose distance is closer to $L_{\xi }$.

**Figure 30:**
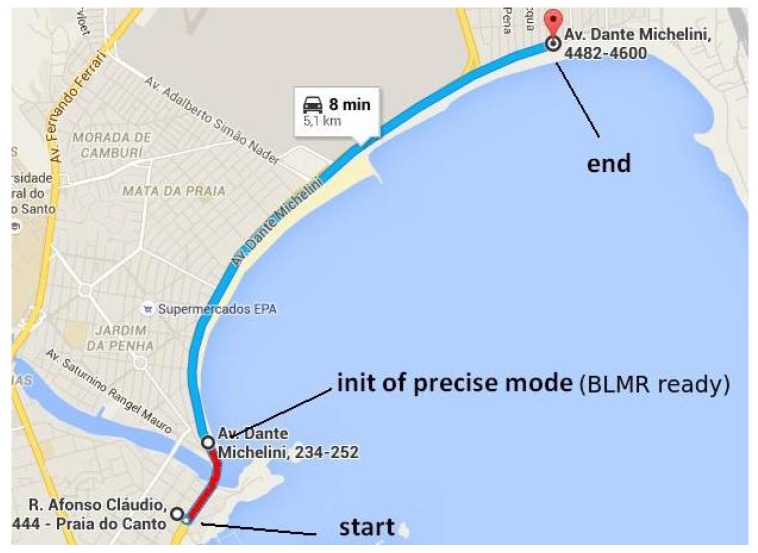
Urban road chosen for the experimental tests.

The protocol for calculating the accuracy is described as follows: (i) first, a trip was made to collect data and build the map; (ii) then 6 trips, in a total of 26.6 km were made running the online mapping program to collect the data; (iii) the localization program was run on the data set generated by each of the trips to calculate the LETG. The calculation is performed only after the vehicle enter in precise mode (see Figure 30, blue track); (iv) finally, the histogram of the absolute values of LETG is calculated.

The mapping trip and the first two localization trips were made on the same day, under similar lighting conditions (a cloudy day). Two localization trips were made 15 days later, also on a cloudy day. Finally, the last two localization trips were made one month after the first, with clean blue sky. The road mapped, shown in Figure 30, is localized in the city of Vitória, ES, Brazil (S20.2971567, W40.2920133) and has a total length of 5.4 km.

#### Results of Lateral Error of Target Point

**Figure 31:**
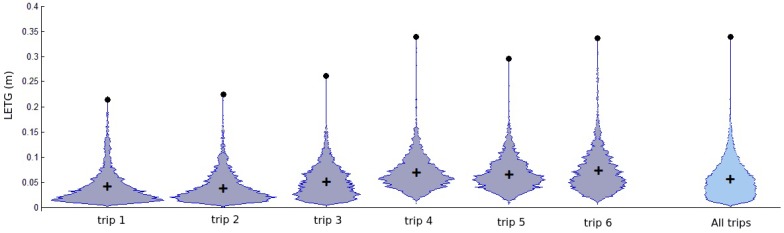
Violin plots (vertical histograms) of the absolute value of the lateral error of the target point (LETG). The crosses indicate the average error, and the dots indicate the maximum error for each trip.

As mentioned before, the accuracy calculation and analysis were performed only after the BLMR was ready. Figure 31 shows the violin plots (vertical histograms) of the absolute value of the lateral error of the target point (LETG), obtained from each individual trip (1–6) and for all trips. Each violin plot represents the error distribution of our autonomous driving variable ξ. In the Figure, the crosses indicate the average error, and the dots indicate the maximum error for each trip. All trips have an average error below 10 cm, and the first two trips resulted in an average error lower than 5 cm. Considering all trips, the average error is 5.7 cm while its value remains under 29.0 cm in 99.9% of the route. For the angular errors, computed according to $\frac{\delta_{\xi }}{L_{\xi }}$, we have $0.13^{\circ }$ and $0.66^{\circ }$ respectively.

These low values of lateral error are an excellent indication that the system is capable of performing autonomous driving, given that, in most cases, a lateral error below 20 cm is sufficient for such applications. This encouraged us to go on the autonomous driving test, presented in the next section.

All the experimental data collected by the test vehicle (maps according to the structure described in Section 5.1) are available in http://bit.ly/1MH1Pqb. Moreover we included a MATLAB script file to access the raw data of the computed LETG so that other kind of statistical analysis can be performed by the interested reader.

### 6.3. Autonomus Driving Tests

Once our localization method demonstrated good accuracy, we started with the autonomous driving tests. Those tests were performed in Italy, and were supported by the laboratory VisLab in the University of Parma. The tests were made with the vehicle named PORTER [26]. PORTER has a servo motor to act on the steering wheel and was modified with the installation of the same hardware architecture of the Brazilian vehicle so that it could run the BLMR localization algorithm. More detailed information is provided in [61].

**Figure 32:**
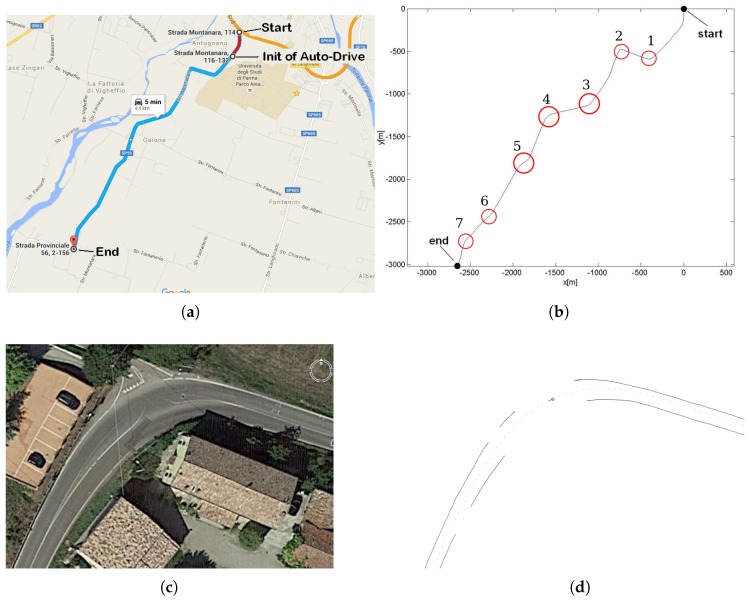
The chosen road section to the autonomous driving tests: (**a**) map from Google Maps; (**b**) map built by the vehicle’s dead reckoning system; (**c**) sky view of curve 2; (**d**) lane markings detected by the vehicle’s vision system in curve 2.

The tests were run on a 4.4 km stretch of narrow country road, illustrated in Figure 32. Two mapping trips were performed, and one of the resulting maps is shown in Figure 32b. The gray line represents the exact path where the vehicle was driven during mapping, i.e., the reference path to be used as a guide when operating in the autonomous driving mode. Figure 32c shows a sky view of curve 2 and Figure 32d the corresponding built map. Because it has a small radius, and the missing lane markings exactly in the middle of it, we considered this curve as the most challenging part for our approach to vehicle localization with low cost sensors, and the autonomous driving algorithm.

We performed a total of 15 autonomous driving tests in the road of Figure 32, in a progressive scale of difficulty: starting with speed of 40 km/h and little traffic and reaching the speed of 60 km/h in more busy hours. In all the trips, the throttle and breaks were controlled manually. From the 15 tests of the autonomous driving, the vehicle completed successfully 14 of them. The sensors and the algorithm demonstrated robustness when faced difficult conditions like sunset lighting on the front windshield glass, failures or absence of lane markings, crossing vehicles on narrow stretches. The only fail occurred in the 11th test, in the curve number 2, when occurred a false detection of the guardrail. It is important to remember that the algorithm presented in this article does not address the detection of moving obstacles, so in some cases human intervention was required to make a light vehicle detour to the track center to divert cyclists or cars parked on the side. All tests were recorded by an internal camera and the camera of the vision system. The complete video can be accessed at https://youtu.be/i9UNKuL5V8E. Other results illustrating the performance of our system, including a comparison with other localization approaches reported in the literature, are presented in [61].

We implemented and tested our system using an Intel Celeron 1.86 GHz based laptop. In such a computer, the lane marking detection algorithm runs in 3.90 ms, the BLMR building takes 0.01 ms, the BLMR pose measurement takes 2.38 ms and the filtering is executed in 0.37 ms. Other parts of the program take about 1.0 ms to run. Therefore, a complete control cycle of our autonomous driving algorithm ran in just 7.66 ms in such a modest computer. Because of that, we consider that our proposed approach is computationally lightweight. Besides that, we also consider that the BLMR algorithm reached this level of computational performance because of the special effort dedicated to every step of the system conception, including the choice of the sensors, the implementation of a customized computer vision algorithm, the choice of a light map structure, the fast map-matching algorithm, and the light filtering algorithm.

## 7. Conclusions and Future Work

This paper presents a low cost sensors approach for accurate vehicle localization and autonomous driving application. The algorithm is based on a light two-level fusion of data from the lane marking sensor, the dead reckoning sensor, and a map. The map is created by the same sensors set, in a manual driven trip. To reach a low cost solution, each sensor module was constructed from standard commercial devices, like USB camera, ordinary GNSS, and MEMs based gyroscope. Special care was taken to overcome the limited performance of standard low cost devices, like the use of near visual information for the camera and, precise temperature control and bias compensation for the gyroscope. The fusion algorithm and map data structure have been designed with special care to be computationally lightweight, so that it can run in low-cost embedded architectures, which contributes to keep the overall system cost low. This is an important issue when thinking about large scale commercial applications. Despite the low cost hardware, the experimental results demonstrated that the system was capable of reaching a high degree of accuracy.

Considering our future work, our main issues can be described as three topics: (i) implementation of the system on a low cost computer; (ii) dynamic correction of the gyroscope bias; (iii) improvement of the filtering.

The good computational performance encouraged us to investigate the viability of implementing the system on a small and cheap single board computer (SBC) or even in a SoC (system on a chip), like Raspberry PI or BeagleBone Black. The substitution of the notebook by a low cost computer system will contribute to reduce the total cost of the localization and autonomous driving system.

Another useful issue to be treated in future are strategies to update the gyroscope bias while the vehicle is in operation. A possible solution is to use the moments when the vehicle stops, for example in a crossing with red traffic light, to make measurements of the bias. Another possible solution, is to use information from the GNSS to correct the bias while the car is moving.

We also believe that the use of a more elaborate scheme of filtering, like Kalman filter, that holds the instantaneous value of the uncertainty for each localization component (lateral, longitudinal, heading) could lead to a more precise and robust estimation of localization. The value of instantaneous uncertainty could be used, for example, to generate some warning signal when the localization accuracy becomes too low for autonomous driving.

Finally, future work also includes the control of acceleration and breaking of the vehicle and detection of obstacles, which are essential for a fully autonomous driving vehicle.
